# Impact of Impact Factor, Publication Audit and the importance of Citations

**DOI:** 10.12669/pjms.324.10927

**Published:** 2016

**Authors:** Shaukat Ali Jawaid, Masood Jawaid

**Affiliations:** 1 Shaukat Ali Jawaid Chief Editor; 2 Masood Jawaid Associate Editor, Pakistan Journal of Medical Sciences, Karachi - Pakistan

Thompson Reuters of United States releases its Journal Citation Report (JCR) every year covering journals with Impact Factor. The JCR for the Year 2016 was released on June 13^th^ 2016 which covered more than eleven thousand journals from eighty one countries which received their Impact Factor. This year the JCR also includes twelve journals from Pakistan including three biomedical journals. In the Year 2011 only seven journals from Pakistan including two biomedical journals were part of JCR. Hence, there has been an increase in the number of journals as regards Indexation in ISI. 1 The medical periodicals covered in 2016 JCR are Journal of Pakistan Medical Association (JPMA), Journal of College of Physicians & Surgeons Pakistan (JCPSP) and Pakistan Journal of Medical Sciences. (Pak J Med Sci). For us it was heartening to note that our Impact Factor had gained more than 100% increase from 0.231 in 2015 to 0.544 in 2016. As such Pakistan Journal of Medical Sciences also emerged as the biomedical journal from Pakistan with the highest Impact Factor. 2-3 Table-I. As regards the number of citations which are much more important, JPMA still maintains the lead with 1,601 citations followed by JCPSP 974 citations and Pakistan Journal of Medical Sciences with 650 Citations. However, it must be mentioned here that Impact Factor is just one of the important parameters and not the only one to evaluate the quality and standard of a journal.^4^-^5^

**Table-I:** Pakistani Journals included in Thompson Reuters Journal Citation Report 2016

No.	Name of the Journal	Total Cites	Impact Factor	Eigen factor Score
1	Pakistan Veterinary Journal	720	0.822	0.001380
2	International Journal of Agriculture and Biology	1,738	0.758	0.002840
3	Pakistan Journal of Botany	3,041	0.658	0.004550
4	Pakistan Journal of Agricultural Sciences	454	0.597	0.000930
5	Pakistan Journal of Pharmaceutical Sciences	1,076	0.581	0.001710
**6**	**Pakistan Journal of Medical Sciences**	** 650**	** 0.544**	** 0.001460**
7	International Journal of Pharmacology	515	0.536	0.000930
**8**	**Journal of Pakistan Medical Association**	** 1,601**	** 0.488**	** 0.002700**
9	Pakistan Journal of Zoology	810	0.478	0.001370
10	Journal of Animal and Plant Sciences	721	0.422	0.002160
**11**	**JCPSP-Journal Coll Physicians & Surg Pakistan**	** 974**	** 0.343**	** 0.002000**
12	Journal of The Chemical Society of Pakistan	670	0.276	0.000970

***Ref.*** *Journal Citation Reports® Science Edition (Thomson Reuters, 2016)*.

It is a pity that at present only three biomedical journals from Pakistan are covered by Thompson Reuters known for its Impact Factor. We are sure some other journals from Pakistan must also have applied but currently it is learnt that first the new journals are accepted in eSCIE (emerging Science Citation Index) which does not have an Impact Factor and after a few years when they are convinced, these journals are upgraded and included in SCIE, Web of Sciences with Impact Factor. Though now it is a bit difficult but not impossible to get included in JCR, Web of Sciences. Efforts should be made to have more and more Pakistani biomedical journals included in the JCR which will also reduce the pressure on the three medical journals which have an Impact Factor. If the journals can ensure regular timely publication, good peer review system to ensure quality of contents, getting into the JCR should not be difficult.

Pakistan Journal of Medical Sciences ever since it was covered by Thompson Reuters for the first time in 2009, has gradually increased its number of citations but its Impact Factor continued to decrease every year from 0.203 in 2009 with 198 citations to 0.098 in the Year 2013 with 336 citations. It was in the Year 2014 that with 410 citations, it improved its Impact Factor to 0.231 and in the current year it further improved by over 100% and achieved an Impact Factor of 0.544 with 650 citations (Table-II). We are determined not only to maintain this but also improve it further for which appropriate steps have been taken. Increase in citations is important to improve Impact Factor and going on PubMed Central has helped us in increasing visibility of the journal which ensures increased citations. 6

**Table-II:** Pakistan Journal of Medical Sciences Citations and Impact Factor (2009-2015).

Year	Total Cites Graph	Journal Impact Factor Graph
2015	650	0.544
2014	410	0.231
2013	336	0.098
2012	349	0.101
2011	283	0.161
2010	275	0.166
2009	198	0.203

We are no more accepting KAP studies, routine survey reports, Case Reports and Reviews are selected very rarely for processing and publication. The emphasis remains on original research, epidemiological studies and Randomized Clinical Trials (RCTs). Manuscripts which are likely to get more citations and the topics which are more relevant to South East Asia and Asia-Pacific Region are given preference. We expect the authors to submit their high quality original research and strictly follow the instructions for authors for the journal on our website to minimize the chances of trauma to their manuscripts and rejection for publication. Processing fee is non-refundable and in case they have any doubt, they are advised to seek prior information before making a submission. Fast Track processing of manuscript is available but only in a few selected cases when the authors have to either appear in FCPS or any other examination. Unfortunately we have noted that there is a tendency on the part of the authors to misuse this facility. This facility, we must reiterate is not for accommodating promotion papers by those who are waiting for their promotion. If this is accepted, then only those who have funds will get their manuscripts published and others will have to wait for too long since there is lot of pressure on limited space available and we can only process a particular number of manuscripts every year keeping in view our financial and human resource constraints. We are indeed grateful to members of the Editorial Board and our valuable team of reviewers who have played a vital role in this achievement of significant increase in Impact Factor and we sincerely hope they will continue to extend their patronage, valuable help, assistance and guidance in this academic activity.

We also do our publication audit every year which enables us to look at our performance.^7^ A critical analysis shows that during the Year 2015, we received one thousand six hundred and three (1603) manuscripts of which three hundred fifteen (315) were published after peer review. One thousand two hundred thirty two were not accepted for further processing because of various reasons, nine manuscripts were rejected because of plagiarism using iThenticate software for screening while twenty seven papers were withdrawn by the authors. Table-III.

**Table-III:** PJMS manuscripts statistics of 2015 at a Glance.

Total Articles Published:	315
Total Articles Rejected:	1232
Rejected because of plagiarism:	9
Articles withdrawn by authors:	27
Under Process:	20

Total Articles Received:	1603

As expected original articles were the largest number of papers published during the Year 2015 followed by Case Reports (21). Table-IV. Majority of the manuscripts accepted for publication during the Year 2015 were from Pakistan (106) followed by Turkey (65), China (65) and Saudi Arabia (32). Table-V. This is contrary to the previous few years when manuscripts from overseas always used to be more than manuscripts from Pakistan.

**Table-IV:** Category Wise Manuscript Published in 2015.

Category	Jan-Feb 2015	Mar-Apr 2015	May-Jun 2015	Jul-Aug 2015	Sep-Oct 2015	Nov-Dec 2015	Total
Original Article	41	41	43	49	45	47	266
Case Report	8	2	2	4	3	2	21
Clinical Case Series	0	0	0	1	0	0	1
Brief Comm.	1	0	0	0	0	0	1
Short Comm.	0	0	0	0	2	1	3
Special Comm.	0	2	0	1	1	0	4
Editorial	1	0	0	1	0	0	2
Guest Editorial	0	0	0	0	0	1	1
Correspondence	0	0	1	0	0	1	2
Review Article	0	2	1	1	3	1	8
Conference Proceeding	1	1	2	0	0	0	4
CME	0	0	0	0	0	1	1
Publication Audit	0	1	0	0	0	0	1

Total	52	49	49	57	54	54	315

**Table-V:** Country Wise Manuscript Published in 2015.

Country	Jan-Feb 2015	Mar-Apr 2015	May-Jun 2015	Jul-Aug 2015	Sep-Oct 2015	Nov-Dec 2015	Total
Pakistan	16	18	14	22	19	17	106
Iran	1	3	1	3	3	1	12
Turkey	13	6	17	10	10	9	65
China	13	13	10	10	10	9	65
Saudi Arabia	3	5	2	9	4	9	32
UAE	1	1	0	0	0	0	2
Romania	1	0	2	0	0	0	3
Malaysia	2	0	3	1	0	4	10
Bangladesh	0	0	0	0	1	0	1
UK	0	0	0	0	1	0	1
Korea	1	1	0	0	2	1	5
Palestine	0	0	0	1	0	1	2
Poland	0	0	0	0	2	2	4
Nigeria	1	0	0	1	1	1	4
Oman	0	1	0	0	0	0	1
India	0	1	0	0	0	0	1
Fiji	0	0	0	0	1	0	1

Total	52	49	49	57	54	54	315

We hope to continue our concerted efforts to further improve the quality of contents and also improve our Impact Factor in the years to come. For this we will need the help, guidance and patronage of our Editorial Board Members as well as valuable Reviewers. We also wish that Pakistan should have more and more journals in general and biomedical journals in particular included in the Journal Citation Report, Web of Sciences by Thompson Reuters as it also projects good image of the country.

In order to help other journals improve their standard, we have been playing our role by organizing training courses for Editors of Medical Journals. So far we have organized five such courses one at PMA House in Karachi, two at University of Health Sciences in Lahore, one at Pakistan Institute of Medical Sciences, Islamabad and one at Khyber Medical University Peshawar. We will be too glad to extend any help and assistance to other journals. One such humble effort is to initiate Dr. Maqbool H. Jafary Memorial Scholarship for Junior Editors from this year. This has been decided to pay homage to the invaluable contribution of our founding Chief Editor Dr. Maqbool H. Jafary who passed away in March 2014. The selected candidates will be provided a stipend money of Rs. 25,000/- (Rupees Twenty Five thousand only) but they will have to arrange their own boarding and lodging for a week in Karachi either by themselves or sponsored by their respective journals. This will be an intensive week long course wherein the selected candidates will be provided comprehensive training in medical journal publishing starting from onsite journal submissions, copy editing, setting up a peer review system, how to grow and maintain the reviewers database, screening for plagiarism using iThenticate software, acquaintance with Digital Object Identifier (DOI), page make up, generating PDF files, functioning of the Open Journal System and details about printing procedures and technology. The objective is that after completing this course, these candidates will put into practice the knowledge and experience gained to improve their respective journals. Every year we intend to train one Junior Editor and hope to put this into practice starting this year hopefully from September-October 2016. The dates for the weeklong course (Monday to Saturday) will be decided after mutual consultation with the selected candidates.
